# Digital Transformation in Learning Organizations

**DOI:** 10.1007/978-3-030-55878-9_14

**Published:** 2020-07-15

**Authors:** Christian Helbig, Sandra Hofhues, Marc Egloffstein, Dirk Ifenthaler

**Affiliations:** 1 Learning, Design and Technology / UNESCO Deputy Chair of Data Science in Higher Education Learning and Teaching, University of Mannheim / Curtin University, Mannheim / Perth, Germany; 2 Department of Education and Social Sciences, University of Cologne, Köln, Germany; 3 Learning, Design & Technology, University of Mannheim, Mannheim, Germany; 4 Human Sciences Faculty, University of Cologne, Cologne, Germany; 5 University of Cologne, Cologne, Germany; 6 University of Mannheim, Mannheim, Germany; 7 Learning, Design and Technology, University of Mannheim, Mannheim, Germany; 8 UNESCO Deputy Chair of Data Science in Higher, Education Learning and Teaching, Curtin University, Perth, WA Australia

**Keywords:** Digital transformation, Learning organization

## Abstract

This concluding chapter takes a summarizing look at the contributions of the anthology, guided by two overarching questions: What dimensions are involved in the digital transformation of learning organizations? Which design perspectives can be used for digital transformation in learning organizations? In conclusion, this chapter leads back to the starting point of the anthology: the project #ko.vernetzt and the question of what significance the dimensions and design perspectives of digital transformation have in learning organizations.

## Introduction

In the light of the title of this book, *Digital Transformation in Learning Organizations*, the demands on digital practices due to the spatial changes in work as well as in learning and teaching present themselves as a new thrust in the discussion about digital change in organizations. For instance, the increasing number of web conferencing tools in organizations during the COVID-19 pandemic is a phenomenon the extent of which cannot yet be predicted. Future studies will show how sustainable the rapid developments in the context of digital technologies in organizations are or whether they are only an expression of a state of emergency. However, as Grünberger and Szucsich (Chap. 10.1007/978-3-030-55878-9_11 in this volume) emphasize, the COVID-19 pandemic also shows the necessity of integrating aspects of environmental and climate protection into processes of digital transformation of organizations.

This anthology was produced in the final phase of the #ko.vernetzt project and contains 13 chapters contributing both perspectives from the project (Part I) and international perspectives (Part II) on digital transformation of learning organizations. The contributions provide indications of the complexity of the perspectives on digital transformations in learning organizations and the dimensions required for the theoretical and empirical capture of digital transformation processes. This concluding chapter attempts to systematize the several and sometimes heterogeneous positions from the individual contributions and elaborate the theoretical approaches.

Thus, the following questions arise at the conclusion of this volume: What dimensions are involved in digital transformation of learning organizations (cf. Section 2)? Which design perspectives can be used for digital transformation in learning organizations (cf. Section 3)? This final contribution tries to first find cursory answers to these questions without claiming to be complete or to be a theorization. The aim is rather to emphasize the points of intersection of the contributions. Finally, the perspectives are linked to the case and project of #ko.vernetzt, which provided the context for this volume (cf. Section 4).

## Dimensions of Digital Transformation in Learning Organizations

The book title raises various questions in connection with the so-called digital transformation. These are fundamental questions, as long as they refer to the transformation of society in the context of its sociality and digitality. They also address very concrete questions in connection with (multiple) single cases, which evoke different concepts and measures in specific organizational contexts. Thus, organizations are also affected by various developments and demands of society. They react to them in several ways described throughout the book.

One of the main questions entangled in the volume is of how digital transformation in learning organizations is to be understood. As many contributions show, the work of Argyris and Schön (1978) remains fundamental to theoretical and empirical perspectives on the digital transformation of learning organizations. Accordingly, “organizational learning occurs when members of the organization act as learning agents for the organization, responding to changes in the internal and external environments of the organization by detecting and correcting errors in organizational theory-in-use, and embedding the results of their inquiry in private images and shared maps of organization” (Argyris and Schön 1978, p. 29). The contributions collected in this volume reference to different dimensions of digital transformation which are linked to several theories of learning organizations. At least four dimensions can be identified as follows:

- **Technical changes in organizations.**

Considering the contributions of Barabasch and Keller (Chap. 10.1007/978-3-030-55878-9_7 in this volume), the first dimension of digital transformation in organizations presents itself as a technical object. Thus, development issues in organizations often arise in connection with the implementation of technologies that are expected to increase efficiency and effectiveness. Here, theories of neo-institutionalism offer perspectives on collective rationality in organizational fields, and isomorphisms can provide explanatory models for such phenomena. The theoretical approach explains structures and modes of operation of organizations by referring to norms, expectations, and concepts of the institutional environment (DiMaggio and Powell 1983).

(b) **Changes in routines and practices.**

The implementation of technologies does not necessarily lead to the initiation of development processes in all cases of learning organizations. This leads us to the second dimension: As Dörner and Rundel (Chap. 10.1007/978-3-030-55878-9_4 in this volume) have elaborated in detail, previous practices must become obsolete in order to initiate educational processes. This “crisis” leads to educational processes in which routines as well as social and cultural orientations are questioned by the actors of the organization and the organization itself. Zeuner (2020) states that educational discourses in the context of crises often understand education as an instrument for maintaining economic growth and employability. However, if education is seen as an integral part of social development, it complements and supports politics and society by helping to shape and influence them (Zeuner 2020). In their contributions, Iovinelli and Elkordy (Chap. 10.1007/978-3-030-55878-9_12 in this volume) and Schiffbauer and Seelmeyer (Chap. 10.1007/978-3-030-55878-9_8 in this volume) show ways in which the implementation of new technologies, education, and the change of action practices in organizations are linked and can be put into practice.

(c) **Technologies as a learning medium.**

The third dimension of digital transformation in learning organizations is represented by digital technologies. They are often described as a medium of and for learning, thus promoting new skills and practices. In this context, reference should be made to the international discourses on digital and media competence, such as the DigComp Framework (Carretero et al. 2017). Here, however, critical aspects of the discourse on media competences also become apparent, e.g., an instrumental shortening (Altenrath et al. 2020, in press). Therefore, the third dimension can be linked with the first and second dimensions of digital transformation in learning organizations, but not necessarily depending on the methodological approach of the articles. Following the contributions of Barabasch and Keller (Chap. 10.1007/978-3-030-55878-9_7 in this volume) as well as Cattaneo, Bonni, and Rauseo (Chap. 10.1007/978-3-030-55878-9_10 in this volume), this dimension can be connected to perspectives of instructional design (Ifenthaler 2017).

(d) **Technologies as consulting and decision-making tools.**

The fourth dimension is related to the increasing possibilities and use of data and algorithms. The contributions of Meier and colleagues (Chap. 10.1007/978-3-030-55878-9_5 in this volume) as well as Berisha-Gwalowski, Caruso, and Harteis (Chap. 10.1007/978-3-030-55878-9_6 in this volume) show that the use of cyber-physical systems and smart machines holds potential for improving learning activities of individuals in organizations and for the development of organizations themselves. Here, digital technologies present themselves as consulting and decision-making tools that can have a decisive influence on the direction of digital transformation in learning organizations. Similar developments can be seen in other fields of education, for instance, Ifenthaler, Mah, and Yau (2019) provide insights into opportunities of learning analytics in the field of higher education which leads to organization-wide change processes (Ifenthaler 2020; Ifenthaler and Gibson, in press).

In summary, the connection of the four dimensions can be differently pronounced. While digital transformation in most cases requires the implementation of technologies, technologies as consulting and decision-making tools are still scarce.

## Theoretical Perspectives on Digital Transformations in Learning Organizations

The digital transformation of organizations implies different dimensions, which make their immediate design and further development from a perspective of research and practice unequally challenging (see Chap. 10.1007/978-3-030-55878-9_2 in this volume). If one observes the developments in detail, one can identify different approaches to the field of the development of organizations at the same time. They are usually theoretically founded, so that the research process and the possibilities and impossibilities of individual or organizational development can be derived from this basic understanding. The contributions of this volume have shown that the development of organizations is often pursued based on a common concern. But the perspectives differ: for example, to which extent research in practice intervenes with and through research, and in what manner assumptions of effects are made? This makes it important for us to accentuate the particular theories that are related to the learning of organizations.

The contributions of Iovinelli and Elkordy (Chap. 10.1007/978-3-030-55878-9_12 in this volume); Cattaneo, Bonini, and Rauseo (Chap. 10.1007/978-3-030-55878-9_10 in this volume); Kowch (Chap. 10.1007/978-3-030-55878-9_9 in this volume); and Schiffbauer and Seelmeyer (Chap. 10.1007/978-3-030-55878-9_8 in this volume) as well as the contributions from the project #ko.vernetzt (Bröckling, Behr & Erdmann, Chap. 10.1007/978-3-030-55878-9_1 in this volume; Helbig, Hofhues and Lukács, Chap. 10.1007/978-3-030-55878-9_2 in this volume; Egloffstein and Ifenthaler, Chap. 10.1007/978-3-030-55878-9_3 in this volume) show that learning organizations continue to depend on the human actors in the respective organization, even in the context of digital transformation. The contributions include different theoretical perspectives on development and design aspects around learning organizations. Although these perspectives are not exclusively linked to digital transformation, the significance of the individual perspectives is demonstrated in connection with digital technologies.

### Individual Participation and Organizational Change

Participatory approaches to organizational development are not new. However, the contributions by Schiffhauer and Seelmeyer (Chap. 10.1007/978-3-030-55878-9_8 in this volume); Bröckling, Behr, and Erdmann (Chap. 10.1007/978-3-030-55878-9_1 in this volume), and Helbig, Hofhues, and Lukács (Chap. 10.1007/978-3-030-55878-9_2 in this volume) show that participatory approaches are gaining importance in digital transformation processes in organizations. Participation, understood as the involvement of as many different actors from the organization as possible, has the purpose of increasing motivation for change and reducing anxiety. Participation also serves to incorporate the specifics of the organizational fields, for example, educational organizations (Helbig, Hofhues, and Lukács, Chap. 10.1007/978-3-030-55878-9_2 in this volume) or social welfare organizations (Schiffhauer and Sellmeyer, Chap. 10.1007/978-3-030-55878-9_8 in this volume), and the specifics of the particular organization itself into the development processes. Approaches and methods of design thinking and human-centered design (HCD) are being increasingly established here. However, questions of decision-making and hierarchies and the assumption of responsibility continue to arise, especially in complex and networked organizations.

### Leadership between Professionalization and Strategy

Both Iovinelli and Elkordy (Chap. 10.1007/978-3-030-55878-9_12 in this volume) and Kowch (Chap. 10.1007/978-3-030-55878-9_9 in this volume) stress leadership as a core area of digital transformation. Kowch places particular emphasis on innovations, informal networks, and experiments. Models such as digital leadership in education (Sheninger 2019) can be connected to this. Overall, these leadership models illustrate a changed understanding of leadership in the context of digital transformation. The new understandings take into account that, on the one hand, knowledge and practices are becoming increasingly differentiated and expert knowledge is becoming more fragmented, while, on the other hand, knowledge and practices are becoming obsolete more quickly and must be renewed. Cattaneo, Bonini, and Rauseo (Chap. 10.1007/978-3-030-55878-9_10 in this volume) follow on from this argument and focus on the development of new professional groups and their professionalization. The example of the “ digital facilitator” shows that digital transformation in educational organizations is increasingly dependent on specialized knowledge that can be expected neither from IT experts nor from education experts. In the future, both new personnel requirements and empirical questions will arise (Ifenthaler 2018).

### Resistance and Inertia

As an important perspective on digital transformation in learning organizations, Scholkmann (Chap. 10.1007/978-3-030-55878-9_13 in this volume) highlights resistance to change. The author emphasizes in the tradition of Argyris (1993) and Kotter (1995) that individual resistance is only one aspect and that both organizations and organizational fields can offer resistance to change. Initial solution options can be found in the previously mentioned contributions. From the perspective of learning organizations, however, further research questions arise on the phenomena of resistance in the context of digital transformation. Does such a resistance necessarily lead to organizational inertia? What are the positive aspects related to organizational resistance, and what potentials does it provide?

## Considerations to #ko.vernetzt

The challenge is still to transfer single concepts and measures to a specific case. The specific case that motivated us to edit this volume was the project #ko.vernetzt and within it a specific educational organization. The project has tackled different issues, which are all located between research and either practice or application in the field of digitization, digital learning, and digital transformation.

What became clear with reference to Argyris and Schön (1978) is that there is technical change in an exemplary analyzed organization. We have observed the change of routines as well as the change of concrete practices. They have also been quantified and described through various surveys. With regard to the role of technology, our research has confirmed different assumptions. However, it has also allowed various interpretations, which were based on the different assumptions of our research in the methodological paradigms. Results have stimulated each other. It became evident that digital technology has one function in the management of an organization. They sometimes occur as decision-making tools.

The contributions in this volume offer various readings of how the project #ko.vernetzt can and should be included in the discourse on learning organizations. The focus is on the relationship between the individual on the one hand and the organization on the other – a relationship that is also understood as subjectivation. Subjectivation is here reduced neither to an event of unfolding, development, or self-construction nor to mere socialization, but must be understood as a constitutive interlocking of foreign and self-reference. Subjectivation therefore refers to the process of learning to lead one’s own life under the leadership of others and to oneself in other peoples and worlds’ relations. In this understanding of subjectivation by Butler (1990), research questions mainly focus on the processes in which people in learning organizations and in the context of digital transformation are made subjects by others as well as themselves. Other research questions have also been generated in the sight of the discussions of leadership. They were condensed through digitalization. Resistance, whether to learning or to organizational change, is also a constant topic in research literature on the learning organization. Thus, #ko.vernetzt with the educational organization involved proves to be a quite typical case.

All findings feed the discourse, but the question is how they can also lead to the development of practice. We assumed on a meta-level that the interlocking of findings and their reflection in the practice of the educational organization would have implications for later action in the organization. The contributions provide various insights into the extent to which research results lead to changes in practice and what kind of participation is possible in the organization. However, the visible differences prove to be particularly productive for the learning organization if they enable themselves to reflect on findings and place them in the context of their own organization. With research projects such as #ko.vernetzt, it is therefore not a matter of working out precisely fitting results for a direct transfer into action mechanisms and management requirements, but rather of creating a social space for reflection on the development of practice, which can only be created through research-based approaches to practice. Accordingly, this volume also emphasizes that digital transformation of learning organizations must be reflected on different levels. In addition to technical issues, they include social aspects as well as the field of leadership. In short, organizations become learning organizations if they put themselves in a position to reflect. This was a continuous mantra of the project #ko.vernetzt.
